# Sleep disturbance and brain health in professional association footballers

**DOI:** 10.3389/fspor.2025.1713730

**Published:** 2026-01-29

**Authors:** N. E. Howarth, M. A. Miller, J. Batten, C. Ji, H. Dawes, M. J. Grey, A. J. Pearce, A. J. White

**Affiliations:** 1 Public Health and Sport Sciences, University of Exeter, Exeter, United Kingdom; 2 Warwick Medical School, University of Warwick, Coventry, United Kingdom; 3 Department Sport, Exercise and Health, University of Winchester, Winchester, United Kingdom; 4 School of Sport Exercise and Health Sciences, National Centre for Sport and Exercise Medicine, Loughborough University, Loughborough, United Kingdom; 5 Swinburne Neuroimaging Facility, Swinburne University of Technology, Melbourne, VIC, Australia; 6 Brain Health Department, Professional Footballers’ Association, Manchester, United Kingdom

**Keywords:** concussion, current players, former players, sleep, sleep quality

## Abstract

**Introduction:**

Sleep is a critical component of normal bodily function as well as for peak performance in athletes. This study examined the associations between quality and quantity of sleep, sleep disturbances, head injuries, and headaches in current and former association football (soccer) players in the United Kingdom (UK).

**Methods:**

An anonymous online cross-sectional survey was sent to members of the Professional Footballers' Association with questions relating to sleep, concussion history, and headaches.

**Results:**

The survey was completed by 600 participants (18 women, 582 men; aged 17–92 years, mean age 44.12 years ± 15.33 SD), comprising 195 current and 405 retired players. Significant clinical sleep disruption was reported in 17.0% of participants, with 22.5% achieving six hours or less of sleep per night. Odds ratios (OR) demonstrated that sleep disruption was associated with headaches [2.66 OR, 95% Confidence Interval (CI): 1.61–4.38; *p* < 0.001], suspected concussions (1.84 OR, 95% CI: 1.03–3.36; *p* = 0.040), and short sleep (4.06 OR, 95% CI: 2.56–6.44; *p* < 0.001). Both diagnosed and suspected concussions were significantly associated with waking up feeling tired or worn out (diagnosed: *p* = 0.019; suspected: *p* < 0.001).

**Discussion:**

A large proportion of former and current footballers experience clinically significant sleep disturbance, which is significantly associated with headaches and concussion history. Further research is needed to explore the long-term relationship between brain health and sleep in this population.

## Introduction

Sleep plays a critical role in neuroprotection and overall health, with poor sleep linked to various adverse outcomes including cognitive impairment, neuroinflammation, and neurodegenerative disease (1). Investigation into athletes' sleep and brain health is emerging but requires further research and attention.

Adults are recommended to have seven to nine hours of sleep (2). Sleep quality is equally important; for sleep to be considered restful, there should be minimal waking during the night (no more than once), rapid return to sleep within 20 min if woken, falling asleep within 30 min, and feeling energised upon waking (3, 4). Both quantity and quality are vital for brain health. Neurophysiological studies suggest that sleep disruption following concussion may involve changes in sleep architecture, including alterations in rapid eye movement (REM) and non-REM sleep stages, which can impact cognitive and emotional processing (5).

Disrupted sleep—whether through poor quality sleep caused by sleep disorders or other disruptions, or through shortened duration due to insomnia or reduced sleep opportunity—can adversely affect brain health. For example, obstructive sleep apnoea (OSA), which partially or completely restricts airflow during sleep, disrupts the glymphatic system and increases neuroinflammation (6). OSA has been linked to increased risk of neurodegenerative diseases (6, 7). Insomnia, characterized by prolonged sleep initiation and difficulty maintaining sleep, is associated with increased risk of dementia, anxiety, and depression (8–12).

Sleep deprivation is linked to poorer cognitive outcomes affecting attention, working memory, long-term memory, decision-making, response inhibition, and visuomotor and verbal functions (13). Moreover, sleep durations under six hours per night have been associated with increased risk of diabetes, hypertension, cardiometabolic disease, and all-cause mortality (14). Emerging evidence also implicates autonomic nervous system dysregulation and neuroinflammatory pathways in the link between sleep disruption and brain injury (1, 15).

Good quality sleep is vital for sport performance, injury prevention and recovery, decreased illness risk, and promoting athletic development (16, 17). Sport-related concussion can impact sleep, often initially increasing sleep duration but with disturbances that may prolong recovery (18, 19). Hoffman et al. (20) found that college athletes with greater wake time and disrupted sleep had longer recovery and more symptoms. Similarly, repeated concussions correlate with heightened sleep disturbances and increased headaches, mood changes, and cognitive dysfunction (21). Electroencephalographic changes during sleep post-concussion further correlate with recovery trajectories, supporting models of bidirectional interaction between sleep disturbance and neurocognitive impairment (1, 5, 22).

Headache is the most frequent concussion symptom and commonly part of post-concussive syndrome (23, 24). Neuroinflammation from brain injury may underpin this association (15). Headaches also correlate with sleep disturbances such as circadian dysthymia and insomnia, which are similarly associated with concussion (25–27).

Professional athletes experience increased sleep disruption largely due to stress, travel, and playing schedules (16, 28). Poor sleep in adolescence and early adulthood may predict longer-term sleep problems (29). High levels of sleep disruption are reported among both current and former professional athletes, potentially impacting on their long-term health. However, there exists limited research specifically examining sleep patterns in retiring and retired athletes, especially in comparison with the general population and with attention to factors influencing sleep. There is growing recognition of the need for longitudinal studies to better understand the chronic effects of repetitive head impacts on sleep and brain health (30).

Like other athletes, association football (soccer) players are at increased risk for sleep disruption (28). Kilic et al. (32) found sleep disturbances in 15.8% of current and 11.0% of retired footballers. Patient report outcome measurement information system (PROMIS) assessments reported sleep disturbances in 28 of 220 former footballers (33). Current footballers also show insufficient sleep duration and symptoms like insomnia and daytime sleepiness, with 39 of 111 participants sleeping less than seven hours per night (17). Among 210 male professional footballers, insomnia severity was associated with greater stimulant use, pre-sleep arousal, and worry/rumination, mediating the insomnia relationship (34). Fernandes et al. (35) found that retired professional footballers had poorer sleep quality than age-matched UK population controls despite a higher prevalence of optimal sleep durations.

In this study, members of the Professional Footballers' Association (PFA) were surveyed to provide insight into current and former footballers' sleep and brain health. The primary aims were to determine the prevalence of sleep disturbances and variations in sleep duration among these athletes, to examine associations between concussion history and clinically significant sleep disturbances, and to investigate the relationship between concussion(s) and headache prevalence. Additionally, potential differences between current and former footballers were explored to better understand modifiable factors affecting brain health and recovery.

## Methods

### Design

This is a cross-sectional survey which has assessed a players demographics, sleep, concussion history. The survey was designed to gain an insight and understanding into sleep and concussion across the different footballing contexts and whether players were retired or playing to develop greater knowledge in this area (36, 37). The design incorporated previously variated tools to increase reliability). In the survey participants have responded about their sleep over the last month and concussion history over their career.

### Participants

Female and male volunteers were invited to participate via the PFA membership mailing list as part of routine communications to its members. All members of the PFA, both current and former players, were eligible to participate. To be included they had be a current member of the PFA which means they were a former or current professional football and had to complete all the questions; there was no exclusion criteria in responses unless they were incomplete. Respondents included 18 women (3.0%) and 582 men (97.0%), the playing level was split across with women competing in the Women's Super League and FA Women's Championship, and men spanning multiple playing levels including Premier League, Championship, League One, League Two, National Leagues, and retired categories (see Table 1). The survey also collected information about duration of career. The biological sex distribution is reported here to contextualise analyses, given the relatively low number of female participants which influenced subset analyses.

**Table 1:** Demographic characteristics of the cohort by sex and current playing level.

Playing Category	n (% within group)	Age, mean (SD)
Women	18	28.06 (4.75)
Women's Super League	15 (83.3)	27.40 (4.66)
FA Women's Championship	3 (16.7)	31.33 (4.51)
Men	582	44.62 (15.27)
Premier League	6 (1.0)	27.00 (6.78)
Championship	40 (6.9)	27.18 (5.54)
League One	31 (5.3)	26.77 (5.64)
League Two	46 (7.9)	27.59 (5.70)
National Leagues	54 (9.3)	29.31 (5.88)
Retired	405 (69.6)	51.94 (12.00)
Playing Females (18)	8.28 (0.46)	0 (0.0)
Total	600	44.12 (15.33)

### Survey

Participants volunteered to complete an anonymous online survey hosted on SurveyMonkey, which was open for a two-week period in March 2023. Recruitment was conducted via the PFA's regular membership email communication (i.e., not a specific request), which included notification of the study and an invitation to participate. Participation was anonymous and voluntary.

The survey included questions covering several key domains including player demographics and playing status, concussion history and knowledge, experience of headaches in the past month, and a sleep disturbance assessment (See Appendix A for details).

Headache frequency was assessed using questions reflecting prior research standards, specifically considering experiencing headaches weekly or more often as the cutoff for analysis [see (38)]. Sleep disturbance was assessed using the Jenkins Sleep Scale (JSS), a validated four-item questionnaire measuring the frequency of common sleep problems over the past month: difficulty falling asleep, waking up several times per night, trouble staying asleep (including early waking), and waking feeling tired or worn out (39). Each item is scored on a six-point frequency scale (0 = not at all, 5 = 22–31 days), yielding a total score ranging from 0 to 20, with higher scores indicating greater disturbance. Consistent with previous large-scale population studies, a cut-off score of ≥12 was used to indicate clinically significant sleep disturbance (40, 41). This threshold has been shown to correspond to the upper percentiles of the general population distribution and is associated with adverse health outcomes, thereby providing a clinically meaningful indicator of significant sleep problems in epidemiological research.

This voluntary online survey was internally reviewed and approved by the PFA. Participation was entirely optional, with no incentives or penalties associated with taking part. All participants, as part of the consenting to take part in the survey, were fully informed that their responses would be used for research purposes by the PFA. Data were stored securely on PFA servers and anonymised prior to transfer for analysis in accordance with data protection regulation and measures. The [redacted] reviewed and approved the data transfer process and secondary analysis of the anonymous data. This was approved by Chair's action dated 12/12/2023.

### Data analysis and statistics

Participant demographic and clinical characteristics were summarised using frequencies and percentages for categorical variables and means with standard deviations (SD) for continuous variables. The JSS results were categorised to distinguish clinically significant sleep disturbances (scores ≥12) from non-clinically significant (<12) based on established thresholds (40). Internal consistency of the JSS was assessed in the current sample using Cronbach's alpha. Further analyses examined associations between sleep disturbance status, concussion history (diagnosed or self-reported), and the experience of headaches occurring weekly or more often.

Logistic regression models were performed using SPSS (version 29.0.1.1, IBM, UK), reporting odds ratios (OR) with 95% confidence intervals [CI; (42)]. Both unadjusted and adjusted analyses were conducted with adjustment for age, biological sex, and current playing status, given their potential influence on sleep behaviour and concussion outcomes.

Subset analyses were conducted for male participants, retired players, and current male players to account for the small proportion of female participants and the heterogeneity of sleep behaviours across sex and playing status groups. A *p*-value less than 0.05 was considered statistically significant. Participants with missing data were excluded from the analysis.

No direct control group was included in this survey; however, sleep disturbance prevalence and other health outcomes are contextualised (see discussion) by comparison to population norms reported in previous studies [e.g., (35, 41)].

## Results

### Participant characteristics

Six hundred professional footballers aged 17–92 years [18 women (3.0%) and 582 men (97.0%)] completed the survey. While the male cohort included both current and former players, all female participants were current players (see Table 1). Women primarily competed in the Women's Super League and FA Women's Championship, and men spanned multiple playing levels including Premier League, Championship, League One, League Two, National Leagues, and retired categories.

### Sleep and brain health outcomes

Table 2 presents results relating to sleep duration (hours), short sleep (six or fewer hours), clinically significant sleep disturbance (JSS ≥ 12), concussion history (diagnosed and suspected), and weekly headache prevalence.

**Table 2:** Sleep, concussion, and headache characteristics across subgroups of the cohort.

Playing category (*n*)	Mean sleep duration h (SD)	Short sleep *n* (%)	Sleep disturbance *n* (%)	Diagnosed concussions *n* (%)	Suspected concussions *n* (%)	Weekly headaches *n* (%)
Women (18)	8.28 (0.46)	0 (0.0)	1 (5.6)	10 (55.6)	13 (72.2)	1 (5.6)
Women's Super League (15)	8.33 (0.48)	0 (0.0)	0 (0.0)	8 (53.3)	11 (73.3)	1 (6.7)
FA Women's Championship (3)	8.00 (0.00)	0 (0.0)	1 (33.3)	2 (66.7)	2 (66.7)	0 (0.0)
Men (582)	7.59 (0.91)	135 (23.2)	122 (21.0)	264 (45.4)	422 (72.5)	95 (16.3)
Premier League (6)	7.67 (0.82)	1 (16.7)	1 (16.7)	5 (83.3)	5 (83.3)	1 (16.7)
Championship (40)	8.00 (0.68)	3 (7.5)	4 (10.0)	18 (45.0)	26 (65.0)	4 (10.0)
League One (31)	8.16 (0.37)	0 (0.0)	0 (0.0)	16 (51.6)	21 (67.7)	3 (9.7)
League Two (46)	8.07 (0.44)	1 (2.2)	3 (6.5)	23 (50.0)	33 (71.7)	7 (15.2)
National Leagues (54)	7.67 (0.75)	9 (16.7)	12 (22.2)	31 (57.4)	40 (74.1)	11 (20.4)
Playing Males (177)	7.93 (0.64)	14 (7.9)	20 (11.2)	93 (52.5)	125 (70.6)	27 (15.2)
Playing Females (18)	8.28 (0.46)	0 (0.0)	1 (5.6)	10 (55.6)	13 (72.2)	1 (5.6)
Playing (195)	7.93 (0.64)	14 (7.2)	21 (10.8)	103 (52.8)	138 (70.8)	29 (14.9)
Retired (405)	7.44 (0.96)	121 (29.9)	81 (20.0)	171 (42.2)	297 (73.3)	69 (17.0)
Total (600)	7.61 (0.90)	135 (22.5)	102 (17.0)	274 (45.7)	435 (72.5)	98 (16.3)

The mean sleep duration in the last month was 7.61 h (SD 0.90). One hundred and thirty-five participants (22.5%) reported short sleep. The mean JSS score was 6.31 (SD 4.89), with 102 participants (17.0%) scoring 12 or greater, a threshold indicative of clinically significant sleep disturbance consistent with prior research (40). The JSS demonstrated good internal consistency in this cohort (Cronbach's *α* = 0.810).

### Concussion and headache prevalence

Of all participants, 435 (72.5%) suspected they had sustained a concussion, while 274 (45.7%) reported a diagnosed concussion (Table 2). Current players were more likely to have a diagnosed concussion compared to former players (*p* = 0.015). Headache prevalence, defined as experiencing headaches weekly or more often, was also reported and examined in relation to sleep disturbance and concussion history (Table 2). Weekly headaches were significantly associated with clinically significant sleep disturbance (*p* < 0.001) and with average sleep duration (*p* = 0.007). No significant difference in weekly headache frequency was found between current and former players (*p* = 0.502).

### Associations between sleep disturbance, concussion, and headache

Logistic regression analyses (Table 3) demonstrated significant associations between sleep disturbance and suspected concussion, weekly headaches, and short sleep, with odds ratios (OR) and 95% confidence intervals (CI) reported for unadjusted and age-adjusted models. Subset analyses for males, retired players, and current male players further explored these relationships.

**Table 3:** Odds ratios (OR) for associations between sleep disturbance and weekly headaches, suspected concussion, and short sleep.

Group/subgroup	Unadjusted OR (95% CI)	Age-adjusted OR (95% CI)
Suspected concussion		
Men and women	1.95 (1.13–3.37), *p* = 0.016	1.91 (1.03–3.36), *p* = 0.021
Men only	2.09 (1.20–3.65), *p* = 0.009	2.04 (1.17–3.57), *p* = 0.012
Playing men	2.57 (0.72–9.18), *p* = 0.146	2.75 (0.76–9.93), *p* = 0.122
Retired	1.96 (1.05–3.65), *p* = 0.035	1.97 (1.05–3.68), *p* = 0.034
Weekly headaches		
Men and women	2.64 (1.60–4.33), *p* < 0.001	2.66 (1.61–4.34), *p* < 0.001
Men only	2.70 (1.64–4.46), *p* < 0.001	2.71 (1.64–4.49), *p* < 0.001
Playing men	5.15 (1.86–14.28), *p* = 0.002	5.03 (1.80–14.03), *p* = 0.002
Retired	2.20 (1.23–3.92), *p* = 0.008	2.20 (1.23–3.92), *p* = 0.008
Short sleep		
Men and women	1.95 (1.13–3.37), *p* = 0.016	1.84 (1.03–3.36), *p* = 0.040
Men only	4.10 (2.60–6.46), *p* < 0.001	4.00 (2.52–6.35), *p* < 0.001
Playing men	5.48 (1.26–18.48), *p* = 0.006	8.01 (2.08–30.87), *p* = 0.003
Retired	3.57 (2.15–5.92), *p* < 0.001	3.57 (2.15–5.92), *p* < 0.001

## Discussion

This study has investigated the prevalence and interrelationships of sleep disturbances, concussion history, and headache frequency among current and former professional footballers in the United Kingdom. The findings demonstrate that clinically significant sleep disturbances are common in this population, with 17% of participants exceeding the Jenkins Sleep Scale threshold. Suspected concussion history was significantly associated with increased odds of sleep disturbance, and weekly headaches were strongly correlated with both sleep disruption and short sleep duration. The results highlight the persistent burden of sleep-related problems in footballers, particularly among those with a history of concussion, and underscore the need for targeted interventions addressing these interconnected issues.

### Sleep and concussion

The mean JSS score in this cohort was 6.31 (SD 4.89), with 17% indicating a clinically significant sleep disturbance (JSS ≥ 12), which is notably higher than the reported 6% in general population samples (41). This aligns with emerging evidence that athletes with concussion or repetitive head impacts experience greater sleep disruption than non-athletes or those without concussion (5, 22). This study extends this understanding by including both current and retired footballers, a group historically underrepresented in sleep and concussion research (28, 30).

Sleep disturbances are strongly associated with suspected concussion history, emphasising the importance of capturing unrecognised or underdiagnosed concussion cases when evaluating brain health outcomes (43, 44). The similar sleep disturbance patterns observed for diagnosed and suspected concussion groups suggest that unrecognised concussions may contribute meaningfully to long-term sleep dysfunction and brain health outcomes, warranting further longitudinal investigation. Specifically, analysis of JSS components revealed that concussion was associated with increased frequency of waking feeling tired or worn out, indicating possible disruption of sleep architecture. This is consistent with neurobiological models implicating neuroinflammation and autonomic dysregulation in post-concussion sleep disturbance (1, 15). Electroencephalographic changes during sleep observed post-concussion further support disrupted sleep physiology as a factor in recovery trajectories (5).

A notable finding is that current players are more likely to have been diagnosed concussions than retired players, suggesting improvements in concussion awareness and diagnosis among active footballers. However, a considerable proportion of both groups suspected concussions without formal diagnosis, highlighting ongoing gaps in detection and management.

### Headaches

Weekly headache prevalence is meaningfully associated with both sleep disturbance and short sleep duration (see Table 3). Headaches are a common post-concussion symptom and frequently linked to sleep disruptions such as circadian dysthymia and insomnia (15, 23, 24, 26). While population-based headache prevalence varies, approximately 23% of the UK population report weekly headaches (38), comparable to the frequency observed here. Globally, prevalence estimates range from 4.6% for general headache disorders to 16.3% experiencing weekly headaches (45). These data underscore a clinically relevant burden of headaches in footballers with concussion history. The findings reinforce the importance of incorporating sleep assessment and management into headache treatment strategies for athletes.

### Strengths and limitations

This study benefits from a relatively large sample size of 600 current and retired professional footballers, the largest study of its kind with this population of elite athletes. The inclusion of both diagnosed and suspected concussion history allows for a comprehensive view of concussion exposure, addressing known under-reporting (44). Furthermore, the use of the validated Jenkins Sleep Scale adds rigour and facilitates comparison with other large population studies [e.g., (40)].

A key limitation is the absence of a direct control group from the general population, restricting causal inference and direct comparison within the study. However, this limitation is partially mitigated by benchmarking the findings from this study against normative data derived from large, representative population samples. For example, Tibubos et al. (41) assessed sleep disturbances using the JSS in a general German cohort of over 2,500 adults, reporting a substantially lower prevalence of clinically significant sleep disturbance (6.0%) compared to the 17.0% observed here. Similarly, Fernandes et al. (35) found retired professional footballers had significantly poorer sleep quality than an age-matched UK population despite a higher prevalence of achieving recommended sleep duration. These comparative data provide an important epidemiological context supporting elevated sleep disturbance in footballers relative to general populations. Although indirect, such benchmarking strengthens the validity of the findings by situating them within established population norms and underscores the clinical relevance despite the absence of a contemporaneous control group. Epidemiological studies have also consistently shown athletes with concussion histories often experience greater sleep disturbances than non-athletes or athletes without concussion (22).

The cross-sectional design limits causal conclusions. Moreover, while the number of female respondents was limited, the inclusion of female footballers provides important insight given the underrepresentation of women in this research area (30). Future longitudinal studies are needed to clarify these relationships and address these limitations (31). Therefore, future studies should encompass sleep diaries over serial and prolong period of time. They should also consider more detailed assessment of sleep over a career and the length of a career. Prospective studies around head injuries and impact in football could also consider sleep immediately after and then serial follow-ups for sleep and symptom assessment.

### Implications

These findings emphasise the need for clinicians and researchers to consider both diagnosed and suspected concussion history when assessing sleep and neurological health in athletes. Sleep hygiene and targeted interventions may play a critical role in managing headaches and improving concussion recovery. Given the complex interplay between concussion, sleep, and headache symptoms, a multidisciplinary approach incorporating sleep assessment should be integral to athlete care pathways.

## Conclusion

This study provides novel insights into sleep disturbance, headache prevalence, and concussion history among current and former professional footballers in the UK. Both groups experience elevated rates of sleep disturbance associated with suspected concussion history and headaches. The findings highlight under-recognition of concussion and suggest a complex interaction between sleep, headaches, and concussion which warrants further longitudinal research to elucidate mechanisms and inform clinical management strategies. Further attention should be given to sleep quality and quantity during competition and training for current footballers.

## Data Availability

The original contributions presented in the study are included in the article/Supplementary Material, further inquiries can be directed to the corresponding author.

## Appendix A—Survey Questions

Participants completed an anonymous online survey containing the following questions:

Demographics & Playing Status

1. Current playing status (Currently playing/Retired)
2. Year of birth (Open-ended response)
3. Total years played football full-time (including scholarship) (Open-ended response)

Concussion History.

4. Have you ever experienced a suspected concussion while playing football? (Yes/No)
  (a) If yes, how many times? (Open-ended response)
5. Have you ever been diagnosed with a concussion from playing football? (Yes/No)
  (a) If yes, how many times? (Open-ended response)
6. Have you ever received education about concussion? (Yes/No)

Headache & Sleep History

7. How often do you experience headaches? (Open-ended response)
8. On average, how many hours of sleep do you have per night? (Open-ended response)
9. In the past four weeks, how often did you:
  (a) Have trouble falling asleep? (Open-ended response)
  (b) Wake up several times per night? (Open-ended response)
  (c) Have trouble staying asleep (including waking too early)? (Open-ended response)
  (d) Wake up after your usual amount of sleep feeling tired and worn out? (Open-ended response).
